# Advances and perspectives in selecting resistance traits against the parasitic mite *Varroa destructor* in honey bees

**DOI:** 10.1186/s12711-020-00591-1

**Published:** 2020-11-27

**Authors:** Matthieu Guichard, Vincent Dietemann, Markus Neuditschko, Benjamin Dainat

**Affiliations:** 1 Agroscope, Swiss Bee Research Centre, Schwarzenburgstrasse 161, 3003 Bern, Switzerland; 2 Department of Ecology and Evolution, Biophore, UNIL-Sorge, University of Lausanne, 1015 Lausanne, Switzerland

## Abstract

**Background:**

In spite of the implementation of control strategies in honey bee (*Apis mellifera*) keeping, the invasive parasitic mite *Varroa destructor* remains one of the main causes of colony losses in numerous countries. Therefore, this parasite represents a serious threat to beekeeping and agro-ecosystems that benefit from the pollination services provided by honey bees. To maintain their stocks, beekeepers have to treat their colonies with acaricides every year. Selecting lineages that are resistant to infestations is deemed to be a more sustainable approach.

**Review:**

Over the last three decades, numerous selection programs have been initiated to improve the host–parasite relationship and to support honey bee survival in the presence of the parasite without the need for acaricide treatments. Although resistance traits have been included in the selection strategy of honey bees, it has not been possible to globally solve the *V. destructor* problem. In this study, we review the literature on the reasons that have potentially limited the success of such selection programs. We compile the available information to assess the relevance of selected traits and the potential environmental effects that distort trait expression and colony survival. Limitations to the implementation of these traits in the field are also discussed.

**Conclusions:**

Improving our knowledge of the mechanisms underlying resistance to *V. destructor* to increase trait relevance, optimizing selection programs to reduce environmental effects, and communicating selection outcomes are all crucial to efforts aiming at establishing a balanced relationship between the invasive parasite and its new host.

## Background

The western honey bee, *Apis mellifera*, is one of the most valuable pollinators worldwide [1–3]. Over the last few decades, increased honey bee colony losses have been reported, mostly in the Northern hemisphere [4–6], possibly as a result of a growing number of interacting threats, such as habitat losses, nutritional deficiencies, pesticides, pests and pathogens [7–9].

Among the parasitic threats, the invasive mite *Varroa destructor* is often identified as the main macrobiotic cause of colony losses of *A. mellifera* in many regions [6, 10–14]. This parasite originates from Southeast Asia, and has shifted from its original host, *A. cerana*, to *A. mellifera* at the beginning of the twentieth century, when the latter was imported to the Russian Far East [15, 16]. The parasite rapidly spread around the world due to the globalized trade with *A. mellifera* queens and swarms [17, 18]. On a global scale, only a few areas, including Australia, some regions of Northern Europe and certain islands, are still considered to be free of *V. destructor* mites and, thus, safe from the parasite’s detrimental impact.

*Varroa destructor* is not lethal to *A. cerana* due to the host–parasite co-evolution [19–21]. The reproduction of the parasite is limited to the transient male (drone) brood of *A. cerana*, which restricts the population growth of the mite. In contrast, in *A. mellifera*, the new host, the parasite infests both the drone brood and the more persistent worker (non-reproductive female) brood, which subsequently leads to high infestation levels [22–24]. Thus, a large proportion of the colony is weakened by the feeding [25–28] and pathogen-vectoring activity [29–35] of the mother mite and its offspring. Upon emergence, the infested individuals do not perform optimally or die early, which threatens colony survival and reproduction [36–39].

To prevent colony losses due to *V. destructor* infestations, beekeepers that rear European *A. mellifera* limit the parasitic pressure on their stocks by implementing control strategies. Such strategies often rely on chemical treatments that involve synthetic miticides, organic acids or essential oils [40]. They may also include biotechnical measures, such as the removal of the preferentially parasitized drone brood. Beekeepers can coordinate these actions within the framework of an integrated pest management strategy [41–43]. Strategies based on synthetic miticides are problematic because their residues contaminate hive products [44] and are likely to favor the emergence of resistant lineages of *V. destructor* [45–47]. Although treatments that involve organic acids have proven to be effective and do not leave residues when used correctly, negative side effects on honey bee health have been demonstrated [48]. Due to such problems, a growing number of beekeepers are attempting to reduce their reliance on chemical treatments [49–51], which has highlighted the need for alternative and sustainable approaches to control this parasite, including the selection of honey bee lineages that survive parasite infestations [52]. This selection aims at favoring the expression of traits that enhance colony survival and subsequently reduce the need for human interventions to control the parasite’s population.

The idea of selecting less susceptible colonies emerged shortly after the global invasion of the mite [19, 53–55], following the observation that several populations could survive in the presence of the parasite without treatments, such as sub-Saharan African subspecies of *A. mellifera* [56, 57] and Africanized honey bees [58–60]. The discovery that some European *A. mellifera* populations can survive *V. destructor* infestation [55, 61, 62] also opened up new avenues of using resistance traits for human-mediated selection. Indeed, survival is often attributed to resistance traits, which, by definition, reduce the parasitic load of the host [63]. Tolerance mechanisms, which allow the host to sustain high parasitic loads [63], are also likely to favor colony survival and to be naturally selected [64], but currently their impact remains largely hypothetical. To our knowledge, no selection program includes tolerance traits; thus, such mechanisms are not considered in this review.

**Fig. 1:**
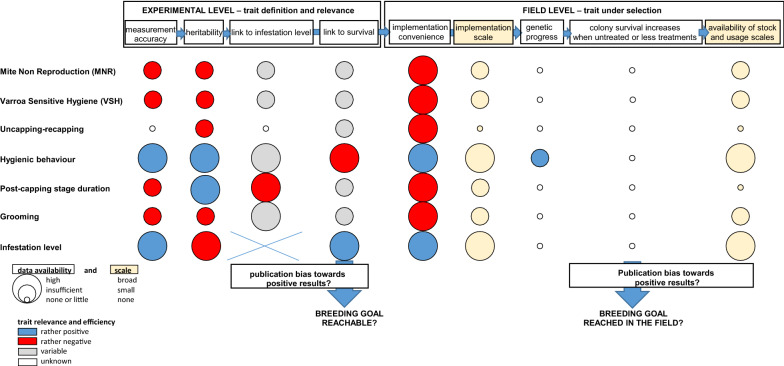
Overview of the theoretical framework known to lead to genetic progress towards a selection objective and to a successful solution to the *V. destructor* problem via selective breeding (above) and evaluation of data availability as well as of the relevance and efficacy of traits under selection for each of the steps towards achievement of the objective (below)

In Europe, numerous resistance selection programs aiming at increasing the frequency of resistance traits in populations started in the 1980s [65], but it has not yet been possible to improve survival of untreated colonies on a broad scale [66]. In North America, lower colony losses of selected lineages (‘Russian’, *Varroa* sensitive hygiene) were recorded [67]. However, high colony losses attributed to *V. destructor* are still reported in the United States [68], which suggests that the current selection strategies have not resulted in a large-scale, sustainable host–parasite equilibrium. Whereas in both regions, knowledge of resistance mechanisms may be increasing [64], a detailed overview of the achievements of past and current selection programs on which to base further progress towards increasing the ability of colonies to survive infestations by *V. destructor* is lacking After nearly four decades of selecting honey bees towards this objective, we expected that sufficient data were available to assess these achievements and to identify to what extent genetic progress has been achieved and the strengths and weaknesses of the selection strategies implemented. Towards this aim, we analyzed scientific peer-reviewed as well as specialized beekeeping literature, which focus on traits linked to survival of infested colonies and on their selection. Our approach consisted of considering whether trait attributes and selection design conformed to the theoretical framework known to lead to genetic progress towards a selection objective [69] (Fig. 1). The factors known to affect selection progress that we considered are: (1) the choice of relevant selection traits, which should provide accurate colony phenotypes, should be heritable and should be linked to the selection objective (i.e., colony survival); (2) the environmental effect that can hinder the expression of heritable traits; and, (3) beyond the theoretical considerations, the practical limitations of selection strategies during field implementation. Finally, we suggest a strategy to improve selection strategies to overcome the obstacles and limitations identified.

### Trait relevance

Several traits observed in naturally surviving populations have been proposed to contribute to the survival of *A. mellifera* colonies infested by *V. destructor* [64, 70]. The expression of some of these traits is thought to lead to the reduction of reproduction and/or survival of the mite within the honey bee brood cell. Others should lead to the reduction of the infestation levels of adult honey bees. The definition of these traits and their evaluation methods are summarized below. The evaluation methods are presented in more detail in Additional file 1.

Mite non-reproduction (MNR) [64], formerly called suppressed mite reproduction (SMR), is quantified by counting the number of viable mated offspring produced per mother mite infesting worker brood [71]. *Varroa* sensitive hygiene (VSH) indicates the ability of adult workers to remove mite-infested brood, for example by quantifying the removal of manually infested brood. Uncapping-recapping measurements reveal the number of cells in which the wax cap was opened and re-sealed by adult workers, which has been associated with colony survival [72, 73]. Hygienic behaviour towards dead brood is used to evaluate the removal rate of frozen or pinned killed brood by adult workers, which has been hypothesized to be a proxy for the removal of *V. destructor*-infested brood [74]. The post-capping stage duration is the amount of time available for the mite to reproduce, and shorter durations are targeted with the aim to reduce the number of viable offspring mites produced by foundresses per reproductive cycle [75]. Grooming refers to the ability of adult workers to remove and potentially wound and kill mites that are infesting them or infesting adult nestmates [40]. Grooming rates are measured in laboratory settings by quantifying the proportion of mites fallen from adult workers and of injured mites. It can also be measured in the field, where, due to the presence of natural mite mortality, only the proportion of injured mites can be recorded. As a phenotype encompassing the cumulated effect of the resistance traits mentioned above, the measure of the infestation level of a colony can be the basis for selection. It can be evaluated through measures of infestation rates obtained by washing mites off adult honey bees or by brood cell inspections [76].

Currently, most selection programs towards improved survival aim at increasing the frequency of single resistance traits. To enable successful selection, trait measurement needs to be accurate—that is, the values obtained should be precise and stable over time to enable a reliable estimation of the genetic value of the colony at any time. Factors internal to the colonies or external to it, (i.e., environmental) can affect this accuracy.

### Measurement accuracy

#### Intracolonial effects on the expression of resistance traits

As the substrate for *V. destructor* reproduction, the quantity of brood produced in a honey bee colony directly influences infestation levels. Therefore, infestation levels can be directly affected by brood interruptions linked to swarming [77, 78]. In addition, fluctuations in brood production may explain, at least partly, why within-colony distribution of *V. destructor* is spatially heterogeneous [79, 80], which affects the expression of VSH: a stronger VSH response is obtained if many infested cells are clustered in a small brood area [81]. Also affected by brood dynamics is the proportion of damaged mites, which is used as a proxy for the grooming ability of a colony [82]. More damage was recorded when the brood was emerging [83], possibly due to mites being more vulnerable to grooming when they are changing their host from emerging workers to nurses [84] compared to when they are adhering to adult workers.

MNR is influenced by the reproductive ability of the mite entering the cell [85–89] and by colony-specific pupal traits [90, 91], the latter effect corresponding to the new definition of SMR [64]. VSH is also triggered in response to brood characteristics, specifically odor cues emitted by infested pupae [92–95] and, possibly, by *V. destructor* [96]. The uncapping-recapping behavior has recently gained attention as a target for selection towards resistance [72, 73, 97]. However, a lack of knowledge about how the factors influence its expression currently limits its use as target trait. For example, its impact on the survival of the mite leaving the cell and hence on her reproductive output differed between studies [98, 99]. Since the mite’s decision to leave its cell once opened is influenced by yet unidentified factors, such as previous disturbance or temperature and humidity, mite reproduction might not be interrupted to the same degree in all populations.

Expression of resistance traits can fluctuate according to the infestation level of the colony, and according to the occurrence of multiply infested cells, its frequency increasing in parallel to infestation levels. The number of offspring per mite, and hence the MNR value, can decrease when infestation increases [100] or be higher in multiply than in singly infested cells [101]. An increasing frequency of multiply infested cells can lead to increased VSH [102, 103]. An increase of VSH was also noted when several infested cells were spatially clustered on the brood frame [81, 98]. A possible explanation for the latter two observations is that the increase of VSH results from increased host damage and/or from the amount of signal triggering the behavior [81, 98]. The influence of infestation levels on the expression of resistance traits is inconsistent and leads to a chicken or egg dilemma, where it is unclear if the infestation level is determined by the expression of a resistance trait or if the expression of a resistance trait is determined by the infestation level.

Because of the natural increase in the infestation levels of colonies over the season, growth rates instead of snapshots of infestation rates are sometimes evaluated [104–106]. However, such evaluations can be biased if infestation levels fluctuate during the season and are not measured at a frequency allowing for the capture of these fluctuations. Decreasing infestation rates in spring or summer have been recorded in a surviving population [107]. The breadth and time of occurrence of the resulting infestation peaks may vary among colonies, even within the same apiary, making it difficult to set an optimal time point to record the infestation and to compare values obtained from different colonies.

The expression of a particular resistance trait can also depend on the expression of other traits. MNR, which is related to the number of offspring by foundresses, was affected by VSH [108–114]. This link may be due to VSH being more frequent when mites reproduce than when they do not [108], and hence selectively removing more fecund mites. However, recent studies did not confirm this biased removal towards cells containing reproducing mites [73, 115]. Yet other studies did not find any correlation between MNR and VSH values at the colony level [116]. These results indicate that the association between traits may vary between populations. Such fluctuating interdependence might result in the selection of a trait not responsible or only partially responsible for increased resistance or survival.

Other pests and pathogens affecting the honey bee colony, and especially its brood, can also affect expression of resistance traits. For example, the occurrence of wax moth larvae can lead to overestimation of uncapping-recapping and grooming behaviours because their feeding activity can trigger honey bee nurses to open and close brood cells [117] and can damage mites [118–120]. Ants can also bias the infestation level estimation stemming from natural mite fall by scavenging fallen mites [121], thereby limiting the accuracy of measurements [121]. In addition, the type of mite-transferred viruses infecting the honey bee populations may influence infestation levels, allowing for or compromising survival. Differences in the virulence of the various deformed wing virus types [122], for instance, may have a direct effect on the number of mites that can be tolerated by the colony [123, 124]. This survival threshold can also be influenced by the haplotype of the mite [125].

The literature focusing on resistance traits indicates that several agents can affect their expression. These agents are the parasite itself, other pests and the host via its biological attributes or via the interaction between resistance traits. As a result, only part of the phenotype measured reflects the ability of a colony to defend itself against the parasite. In addition, the prevalence of these agents is driven by their intrinsic cycles (e.g., seasonal rhythms), thus decreasing the accuracy and reliability of phenotypes. Seasonal rhythms are mostly dictated by environmental factors; thus, next we consider how these factors affect the expression of resistance traits towards *V. destructor*.

#### Environmental effects on the expression of resistance traits

Temperature and humidity are probably the most important environmental factors that affect the expression of resistance traits, as they affect several attributes of the agents described in the previous section. Infestation growth rates recorded early or late in the season have been found to correlate negatively with temperature and positively with relative humidity [126]. In addition, infestation levels depend directly on brood quantities, which can be affected by multiple factors, including beekeeping management (e.g., hive size, colony divisions) as well as food resources and climate [127–129]. Grooming is also affected by climatic conditions [82], with less grooming being performed in spring than in summer [130]. The impact of grooming on mite mortality is generally reduced at lower temperatures [131, 132] and at higher humidity [132], indicating that selecting this particular trait could be insufficiently efficient to reduce the mite population significantly during wintertime, when grooming could have a strong impact since brood is generally absent and all mites are exposed to this behavior. MNR also varies between years [133], potentially due to temperature fluctuations that affect the number of viable offspring produced per female mite [134]. Temperature effects on the duration of the post-capping stage have also been recorded [135]. Marked differences of up to 24 h were observed in relation to this duration within individual colonies, possibly due to the heterogeneity of the brood temperature that drives brood development [136, 137]. The impact of temperature on development also results in strong seasonal variations in its duration: longer development times were measured in late summer or fall when compared to spring [138, 139]. To have a protective effect, the duration of the post-capping stage should be kept short, even under the lower temperatures experienced in fall, which is physiologically unlikely.

The availability of food resources is known to affect the expression of several resistance traits. This is particularly the case for hygienic behavior, which is expressed more frequently when food availability is high [140, 141]. Similarly, rates of mite removal by VSH doubled after colonies were fed with sugar water [141]. Trade-offs between foraging and VSH behavior as well as an effect of the brood to honey stores ratio in the colony on VSH have also been noted [116]. MNR was found to be lower in colonies during periods of greater pollen availability [142]. In times of pollen shortness, brood removal can also occur due to cannibalism, resulting from protein deficiency [143], which biases the measure of VSH.

Human factors, via the evaluation of traits and the management of selected stock, can also decrease the accuracy of trait measurement. The infestation levels of colonies in the late summer result from the initial infestation in the spring, from the fecundity and longevity of *V. destructor* foundresses and from the defence behaviors of the host. However, they can also be significantly affected by the horizontal transmission of *V. destructor* between colonies due to drift or robbing, which depend heavily on beekeeping management [104, 144–150]. Mite influx from neighboring colonies and apiaries is not measured and thus invisible to the evaluator, and hence biases colony phenotyping. Unreliable assessment of the resistance potential of a colony based on its infestation level is particularly likely to occur if mite transfers affect colonies located within the same apiary differently. Indeed, honey bee selection requires that all colonies placed within the same apiary share almost identical environmental conditions, so that differences between colonies can be attributed to genetic differences to a large extent. The heterogeneity of horizontal mite transmission among honey bee colonies [147] is likely confounding the assessment of the impact of resistance traits on the infestation level.

Horizontal transmission is more likely to occur in regions in which colonies are kept at high densities [147] or when the inter-colony distance within the apiary is low [151, 152]. Thus, single colony resistance mechanisms with a significant influence on the infestation level may be more easily detected when horizontal mite transmission is low, such as during the early season or during honey flows. Removing ‘superspreaders’, i.e. colonies above a certain infestation threshold [63], could also be an option to reduce horizontal transmission. However, this approach entails the risk of removing colonies that may start expressing resistance behavior in the near future or potentially tolerant colonies that are unharmed by elevated infestation levels. Infestation thresholds that are critical to colony survival may vary according to environmental conditions and, therefore, do not offer suitable values for guiding selection programs. Their effectiveness would be increased if they were locally determined, thereby avoiding as many biases as possible, although this would involve an extremely tedious process [76, 153, 154].

Human influence beyond beekeeping can also affect selected traits: grooming, for instance, was found to decrease when workers were exposed to the pesticide clothianidin [155]. The same compound was also found to decrease hygienic behavior towards dead brood [156]. Thus, periodical use of this chemical in agriculture can reduce the effectiveness of the defence mechanisms of the colony against *V. destructor* and calls into question whether colonies selected for resistance traits sufficiently express these traits when kept in intensive agro-ecosystems.

Few studies have performed repeated measurements to assess whether and how much the large number of host, parasite or environmental factors affect the expression of resistance traits [116, 157]. Performing such repeated measurements during colony evaluation processes has nevertheless been suggested for different traits to ensure data reliability [61, 116, 157–159]. Indeed, the repeatability of resistance traits is often low: it ranges between 0 and 0.4 for MNR [116, 159] and between 0.21 and 0.33 for hygienic behaviour towards dead brood [116, 159]. Values found for recapping and grooming are in the same range, i.e. 0.35 [159] and 0.48 [158], respectively. Up to five repeated measurements are required to assess the VSH level of a colony [157]. The fact that repeatability is relatively low casts doubt on the reliability of single values and raises the issue of how to aggregate several values when measurements are repeated. Averaging the values does not provide a reliable measurement of the true resistance level of the colony unless the variability originated from imprecise measurement methods, in which case repetition increases accuracy. Variation in the expression of a resistance trait over time indicates that factors yet to be identified influence this expression. Once identified, it becomes possible to correct for their effect. If these factors are not easily identifiable or not identifiable at all, the key periods when resistance traits are likely to have their maximum effect on survival should be targeted (for example, during the production time of winter bees). Traits that can be accurately and reliably estimated do not guarantee success; they also need to be sufficiently heritable for their frequency to be increased by selection.

### Heritability

**Fig. 2:**
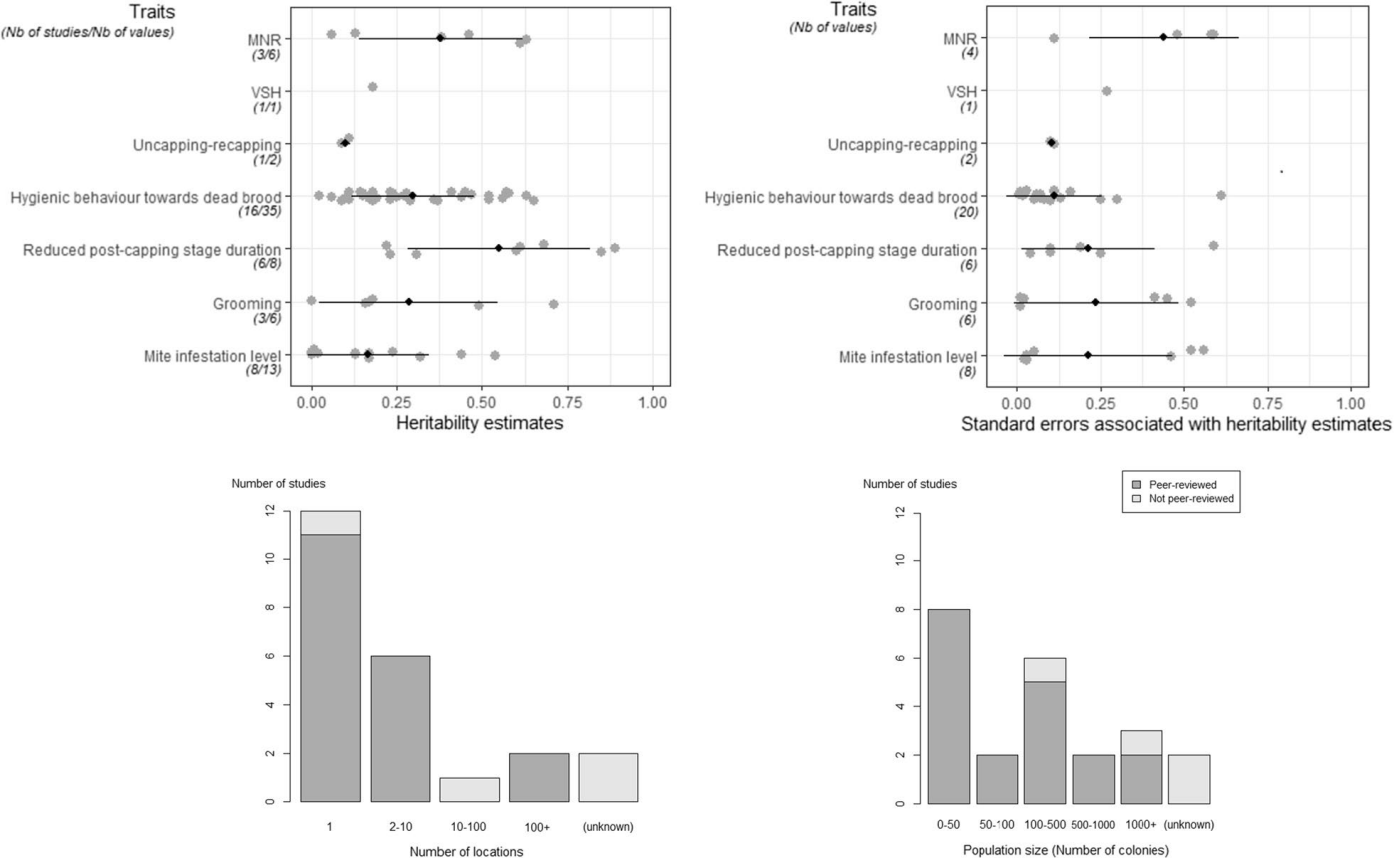
Top-left: Distribution of published heritability estimates proposed for resistance traits mentioned in the literature and Top-right: Distribution of published standard errors associated with heritability estimates (only publications reporting standard errors around heritability estimates were included). Black bars represent the standard deviation from the mean value (black rhombus). Grey dots represent individual values. Bottom-left: Distribution of peer-reviewed and non-peer-reviewed studies according to population size used to assess heredity. All traits were pooled. Bottom-right: Distribution of peer-reviewed and non-peer-reviewed studies according to the number of colonies used to evaluate trait heredity. All traits and locations were pooled

The literature provides variable levels of knowledge about the heritability of each resistance trait. For both VSH and uncapping-recapping, a single heritability estimate is available, which makes it difficult to determine if these traits are relevant for selection towards resistance. In contrast, 16 studies present heritability estimates for hygienic behaviour towards dead brood, which provides more background information to assess the relevance of this trait. However, heritability estimates varied strongly for this and other traits. Heritability of hygienic behaviour ranged from 0.02 to 0.65, which suggests that selecting this trait might not be successful in any environment or population. In addition to variation in heritability estimates, the standard errors were occasionally as high as the estimates themselves (Fig. 2) and see Additional file 2: Table S1. This was especially the case when these estimates were derived from a small number of colonies (< 100), casting doubt on the reliability of their value (Fig. 2) and see Additional file 2: Table S1. Another limitation to their reliability is that many studies recorded traits in a limited number of locations (Fig. 2) and see Additional file 2: Table S1, which hinders our ability to differentiate between additive and non-additive genetic effects, possibly leading to an overestimation of heritability estimates. Issuing general recommendations towards the selection of a particular trait in all populations could be accomplished by comparing heritability values among studies. Unfortunately, this is not feasible because different methods were used to generate the estimates (e.g., regression between parents and offspring or calculation of the ratio between genetic and phenotypic variance) (see Additional file 2: Table S1).

It is worth noting that heritability might be confounded by epigenetic processes. A genetically inherited trait is indistinguishable from a trait acquired via social learning, when workers have the ability to transmit acquired knowledge across generations: thus, behaviours may be expressed by related workers without a genetic causality. Social learning has been identified in other insects, such as fruit flies [160] and bumble bees [161–163], which adapt their behavior after observing conspecifics. It has rarely been investigated in honey bees, which is surprising given that they are a model organism to study learning and memory in insects [164]. One of the few studies on the topic ruled out the social transmission of hygienic behaviour towards dead brood [165], but learning may play a role in other resistance traits against *V. destructor*. For instance, grooming was performed more intensively by workers of naturally surviving *A. m. intermissa* colonies when they were in contact with older workers. Younger nurses could have learned the behaviour from their older nestmates [166]. Such a phenomenon could lead to the loss of resistance if queens from selected lines are introduced into foreign colonies.

Several limitations linked to the heritability estimation procedures may thus decrease the quality of these estimates and hamper progress in the selection for resistance traits. More heritability estimates, based on hundreds of colonies in different locations, should be obtained and published, especially for those traits that gain interest among beekeepers, such as VSH or uncapping-recapping. Screening the heritability estimates for the resistance traits available to date shows that values are often higher than 0.20 (Fig. 2) and see Additional file 2: Table S1 and should therefore lead to increased survivability over time. However, the improvement of colony survival despite infestation by *V. destructor* via selection requires that heritable traits are directly or indirectly linked to a survival mechanism. Next, we review the degree of knowledge on this link and hence on the relevance of the traits that can be selected towards the desired objective.

### Link to survival

The publications from which the relevance of resistance traits to be selected can be deduced are listed in Additional file 3: Tables S2 to S7. This compilation shows that the causal link or association between known resistance traits and infestation level or the survival rate of colonies is not well established. The main reason for this weak link is that most of the studies were performed on treated colonies, which restrains the amount of data available to accurately determine effects in terms of improved colony survival. Another contentious point is that several traits (e.g., VSH, MNR, uncapping-recapping and hygienic behaviour towards dead brood) have only been observed in some naturally surviving populations [64, 70, 115]. Even within a single honey bee population, the contribution of a particular trait to survival can be unclear: in Africanized honey bees, two studies indicated that VSH may reduce infestation level [167, 168], whereas another concluded that it is not a key resistance factor [169] (see Additional file 3: Table S3). This suggests that VSH may have no influence on survival or may only have an effect in the presence of other locally expressed traits or environmental factors in some populations.

Generalization from observations made at the scale of brood cells or adult honey bees to colony phenotype may also be unwarranted. Although high MNR would be expected to decrease colony infestation level, this link was not observed in all populations [170–172]. However, generalization from cell to colony appears warranted in the case of uncapping-recapping, which does not have a direct impact on mite reproduction at the cell level but is associated with colony survival in various populations [72, 73] and thus considered a valuable proxy to identify resistance against *V. destructor* [73].

Generalizing the occurrence of a trait at yet another level, i.e. from workers to other castes in the colony, can lead to suboptimal selection results. Worker instead of drone brood is routinely examined for the expression of resistance traits (e.g., MNR, hygienic behaviour towards dead brood), although trait expression may differ between castes. In such instances, only part of the colony phenotype is captured. Uncapping-recapping of worker brood, but not of drone brood, was reported in naturally surviving populations [73] (see Additional file 3: Table S4). Similarly, frequency of VSH was high towards worker brood but was only low towards the drone brood of the same colonies [173] or vice versa [141]. Thus, the contribution of traits measured on worker brood to reduce the infestation level may be wrongly estimated in colonies rearing many drones (e.g., drone-producing colonies in selection programs).

Another occasionally misleading generalization occurs when results obtained in the laboratory are extrapolated to the field. To date, grooming has been studied primarily in controlled laboratory conditions, where the proportions of damaged mites differed from those obtained in field conditions [174], which leads to misestimating the effects of this trait. This may have contributed to a decrease of interest in selecting for grooming [120, 175–178] (Additional file 3: Table S7).

Similarly, the generalization that hygienic behaviour towards dead brood, which is known to be associated with resistance to chalkbrood and American foulbrood [179–181], will also lead to removal of *V. destructor*-infested brood appears unwarranted, although positive correlations between both traits have been recorded in some publications [74, 158, 182]. First, the value of this trait is often a poor predictor of the colony infestation level [183, 184]. Second, the selection of hygienic behaviour against dead brood did not consistently increase resistance against *V. destructor* (see Additional file 3: Table S5). Several phenomena can explain these observations. Some honey bee lineages selected for hygienic behaviour express a low removal response only against *V. destructor*-infested cells. Positive correlations between the removal of freeze-killed brood and the removal of *V. destructor*-infested brood cells were occasionally found, but only when the brood was infested by two mite foundresses [103, 141] and not when the brood was infested by a single foundress [102, 103]. This may be due to the need for a large amount of chemical stimuli to trigger the removal of dead, diseased or infested brood [92, 185–189]. Thus, hygienic behaviour might only be triggered in cases of high infestation, which may be too late to favour colony survival. These results suggest that selection of hygienic behaviour towards dead brood may not be adapted to select for resistance against *V. destructor* [190] (see SM3-4). It is currently recommended that this trait can be used to pre-select colonies, but that more *V. destructor*-specific traits also need to be applied to ascertain the resistance potential of a given stock [191].

In other cases, the difficulty of relating traits to infestation or survival arises from confounding effects. Here again, grooming can be used as an example when it is evaluated by the number of injured mites fallen from tested colonies. Some deformations of the dorsal shield of the mite are related to the mite’s ontogeny and do not reflect the action of honey bee mandibles [118–120]. Similarly, missing legs on mites can derive from post-mortem decomposition and not from a defensive behaviour of the workers [120]. This may lead to an overestimation of the effectiveness of grooming and to false positives for this trait because of a weak mechanistic link to reduced infestation.

In addition to generalization and confounding effects distorting the links between traits and colony survival, other traits were chosen based on assumptions only. This is the case for the short capping durations. Shorter post-capping durations are assumed to interrupt the reproduction cycle of *V. destructor* and subsequently reduce the number of offspring per founder mite [75]. Several arguments weaken the validity of this assumption. A shorter developmental time may result in more brood cycles per year, thereby counterbalancing the negative effect on individual mite reproduction by increasing the number of generations [40]. To decrease mite fecundity to a level at which the mite population in a colony would be stable over the years, modelling results indicate that a decrease of two days in the post-capping stage duration of workers would be necessary [192, 193]. Even if a reduction of 17% in the duration of the pupal stage could be achieved by selection, physiological effects of parasitism could prolong the duration of the post-capping stage of infested pupae [92, 194] to the point that a reduction generated by selection would be negated [194]. These limits could explain why the majority of the studies concerning the post-capping duration have led to inconclusive results in selection programs (see Additional file 3: Table S6) and highlight the need to take possible side effects or biological costs of a trait into consideration when evaluating its relevance for selection.

The degree of generalization and assumption made when formulating a trait’s link with survival as well as on confounding effects affect the degree of certitude with which this trait can be considered as relevant for selection. The overall variability in the strength of the link between traits and survival recorded in the literature suggests that a major challenge for selection programs lies in defining the most relevant trait to select for in a given population. When this link appears weak, survival of selected populations may be attributable to environmental factors rather than to the increase in frequency of a heritable trait. Next, we review the literature to determine whether survival can be explained solely on the basis of favourable environmental factors.

### Environmental effects on colony survival

In addition to their effect on the expression of several resistance traits (see “Environmental effects on the expression of resistance traits” section), environmental factors can also have major effects on the survival of the *V. destructor*-infested colony independent of the trait selected. This happens because the outcome of the selection program can be the result not only of the accumulation of additive genetic effects via selection for heritable traits (see “Trait relevance” section) but also of the environmental effects and genotype-by-environment interactions. These effects can create the illusion that selected colonies are resistant, while survival is partly or entirely enabled by environmental factors. Honey bees are particularly exposed to highly variable environments compared to other livestock because of their resource acquisition from large areas covering several square km.

### Pathogens

Pathogens belong to the category of environmental factors that affect honey bee health [8, 195, 196]. The survival of some European *A. mellifera* populations was attributed to the occurrence of mite-vectored virus strains of low virulence, which are thought to enable colonies to tolerate more mites. Tolerance to high *V. destructor* infestations was observed in surviving *A. m. ligustica* colonies from the island of Fernando de Noronha, Brazil [197, 198]. It is very likely that this survival is associated with the lower virulence of the Japanese haplotype of *V. destructor* found on the island [125] or of the viruses it vectors [199] and not with host resistance. This idea is supported by the fact that when exposed to different mites (Korean haplotype of *V. destructor*) and different virus types after relocation, these colonies did not show higher survival than local controls [200]. Similarly, *V. destructor*-infested *A. mellifera* colonies in Papua New Guinea and Solomon Islands survive without acaricidal treatments, which could be due to the absence of deformed wing virus (DWV) in these populations [201].The survival of the Gotland population in Sweden was also partly attributed to a tolerance against viral infections [70, 202–204]. Tolerance to viruses could result from natural selection for more virus-tolerant colonies [204] and/or for less virulent viruses [203]. Tolerance to DWV could also be favoured by a resistance to other viruses [202], by decreasing the overall pathogenic pressure on the host. Both tolerance and resistance are likely co-occurring in this population. If natural selection for virus tolerance is confirmed, it may have been facilitated by the isolation of the Gotland colonies, an environmental condition favouring host–parasite co-evolution. This condition can hardly be reproduced in common beekeeping conditions. In Wales, the involvement of viruses in colony survival is hypothesized via the superinfection exclusion of the virulent variant DWV-A by the less virulent variant DWV-B [122, 205, 206]. However, other results suggest a higher virulence of DWV-B when compared to DWV-A [207, 208]. Therefore, the contribution of the virus populations to the observed survival of these colonies remains unclear. Viral loads also fluctuate during beekeeping season [209–212] or following colony migration [213], which can directly influence mite virulence. When virulence of pathogens is low or naturally decreases over time due to natural selection [214], an illusion of selection success can arise.

### Colony size and density

Infestation levels depend on colony size and particularly on the quantity of brood available for *V. destructor* reproduction [64, 78, 215–217]. The quantity of brood produced in colonies is known to be particularly influenced by environmental factors such as climate and food availability [128, 129, 218]. Colonies that are located in areas with low food resources during summer, for instance due to droughts [219, 220], are likely to build up smaller mite populations. As a result, winter honey bees in these colonies should be healthier and risks of colony losses correspondingly lower.

Cavity size can also constrain colony and brood size: colonies nesting in small cavities have less volume available for brood production [78]. The importance of nest size for colony survival was supported when untreated susceptible colonies were kept in small hives and their survival increased to levels comparable to untreated feral colonies surviving in the Arnot Forest (New York, USA) [221]. Small cavities further contribute to survival since they promote frequent swarming and increase air humidity, which are factors known to reduce infestation levels [78, 222].

The spatial dispersal of colonies in their environment also affects their survival chances [151, 223]. In the Arnot Forest, distances between nests often reach hundreds or thousands of meters [224], which reduces the horizontal transmission of the parasite between colonies. Therefore, their survival may not be associated with a genetic resistance [224]. Supporting the idea of a non-genetic survival mechanism, colonies from this feral population transferred to a conventional apiary, in which colonies are kept in close proximity, did not show reduced infestation levels when compared with colonies from susceptible stock [224]. Modelling the effect of colony density and inter-colonial distances on *V. destructor* dispersal [225] differed from the observations in the field [151, 223], highlighting the insufficient understanding of *V. destructor* dispersal.

### Genotype-by-environment interactions

Although genetic background could be excluded in some cases, survival could also be conditioned by genotype-by-environment interactions. Naturally surviving honey bee colonies expressing resistance have often been imported and used as starting material for selection. However, colonies headed by imported queens often failed to show better resistance or survival than local controls. This phenomenon may be due to genotype-by-environment interactions that favour the survival of locally selected colonies. Such interactions can be identified by comparing colonies of a given population kept in their original range with colonies of the same population kept elsewhere under different environmental conditions [226, 227]. Although their designs do not allow conclusions to be drawn on the occurrence of genotype-by-environment, several other studies suggest their involvement and convincingly show high environmental effects on survival. Low survival was observed for naturally surviving colonies imported from South America and South Africa to Europe, or of their hybrids with local European *A. mellifera* [58, 200, 228–230]. Similarly, colonies from the surviving Avignon population in France, relocated to other European countries [70, 227, 231] or to Canada [232] did not show differences in infestation levels or survival rates compared to local stocks. Seemingly better results were obtained when colonies from the ‘Russian’ lineage were imported to Germany [233]. Pure colonies or hybrids with local stock showed lower infestation levels and more damaged mites than control colonies [234]. However, lower infestations could be explained by the lower colony and brood sizes seen in the ‘Russian’ lineage in their new environment and not by a genetic resistance trait [235, 236]. In addition, low survival rates were observed for these imported ‘Russian’ colonies [236]. Therefore, it is likely that the low resistance measured in Germany corresponds to the lack of adaptation to the new environment.

The likely frequent occurrence of genotype-by-environment interactions indicates that adaptation to local conditions plays a major role in colony survival and restrains the possibility to export resistant colonies to regions with different environments. Because of the lack of initial local adaptation, importing resistant colonies from other regions or environments bears low chances of success, and selecting local stock is recommended. Aside from the uncertain performance of the introduced stock, detrimental side effects such as admixture with local populations and the risk of introducing foreign pests and pathogens are problematic [18]. Genotype-by-environment interactions also make the selected colonies susceptible to local changes in environment [237], especially in the current context of increased climatic variations [238], which have direct impacts on plants and pollinators [239, 240]. Therefore, programs aiming at improving colony survival should target resistant traits resilient to environmental changes. In addition to the choice of relevant traits for selection towards better survival and low dependency of survival on environmental factors, a successful selection program depends also on practical constraints.

## Implementation of selection strategies in beekeeping practice

Selecting honey bee lineages that are capable of surviving *V. destructor* infestation would be of little interest for breeders if the implementation of the selection program was impractical, tedious or costly in resources. The biology of the honey bee itself constitutes an obstacle, and the phenotyping of colonies, as shown above, is challenging. Once these obstacles are overcome, the acceptance and use of selected stocks depend on whether they correspond to the beekeepers’ objectives in terms of efficiency to resist to *V. destructor* infestations and of other desirable traits.

### Constraints of honey bee reproductive biology

Compared to other livestock species, selecting *A. mellifera* is difficult due to its reproductive and genetic characteristics. The queen performs nuptial flights across large distances [241–245]. This implies that controlling mating is challenging and requires isolated mating stations to exclude mating with sexuals from unwanted genetic backgrounds [246] or artificial insemination, which allows an even more precise control of drones [247].

The appropriate design of honey bee selection programs that permit the calculation of reliable heritability and breeding values, is crucial in terms of generating and monitoring genetic progress. However, such strategies are difficult to implement due to the complexity of honey bee genetics. As honey bee queens mate with many drones [248–250], a colony, unlike other livestock, is an assembly of worker subfamilies (groups of super- or full-sisters) rather than a single animal. Since only queens (i.e., the dam of the workers) are carried through the selection process, their breeding values need to be calculated from the colony phenotype. Together with multiple mating and the haplodiploid reproductive system [251] of the honey bee, these biological specificities lead to more complex breeding value estimations compared to other livestock species [252–254]. The calculation of reliable breeding values requires knowledge of both animal genetics and honey bee biology and complex algorithms. This complexity may explain why calculation of breeding values and heritability estimates have only been performed in a limited number of programs to date (see Additional file 2: Table S1).

### Field evaluation of resistance traits

An important limitation of selecting resistance traits in the field is that their evaluation is tedious. Selecting for MNR or VSH is time-consuming, as a minimum number of infested brood cells need to be screened in order to generate reliable results [71, 76]. Reaching this number can require the opening of several hundred brood cells. The lower the infestation level, the larger the number of cells that need to be opened. As a consequence, the colonies of greatest interest, i.e. those with the lowest infestation level, are the most time-consuming to evaluate and sometimes cannot be phenotyped at all if the infestation level is too low. To solve this problem, for VSH, the desired number of cells can be manually infested [76], which is also time-consuming and requires maintaining highly infested colonies as mite donors. Therefore, this process can only be applied on a sufficient number of colonies with sufficient workforce. As shown in the first “Trait relevance” and second “Environmental effects on colony survival” sections, obtaining reliable phenotypes can also require repeated measurements, adding to the cost in time and resources, which might exceed what is possible for breeders. In addition to data size, a further limitation is data precision [64]. The methods commonly used to assess traits are insufficiently precise if based on a sample of workers that is too small or on a number of colonies that is too small [255].

The apparent weakness of the links between trait expression and colony survival (see “Link to survival” section), which hinders reaching selection objectives, can originate from variable definitions of the traits under selection. More precise definitions would help to standardize evaluation methods and provide more comparable results. Even when traits are well defined (VSH, MNR, hygienic behaviour towards dead brood, reduced post-capping stage duration, infestation level), their evaluation is often conducted using different methods (see Additional file 1), making it difficult to compare outputs (e.g., phenotypes, phenotyping accuracy, repeatability, link with objective) between populations to obtain a general overview of the relevance of a trait. For example, VSH can be measured as the removal of manually infested cells [115, 182, 256] or by less effort-intensive measures of the changes in infestation rate of brood frames transitorily inserted into highly infested colonies before being returned to the test colonies [157, 186, 257–260]. However, with this method, it is not known whether the infested pupae have been removed (VSH) or whether the mite escaped from the cells opened by the worker (uncapping–recapping). Another method, using photographs of the inserted brood frame before and after being returned to the test colony, has also been used [114, 261]. It allows to determine whether the reduction in infestation measured following the opening of remaining brood cells correlates with brood removal or not. However, this approach does not indicate whether or not the removed brood was infested. Measures of infestation rates are also obtained by a variety of methods (see Additional file 1) that provide different values for this parameter.

Unreliable results may be obtained if the measurement of the trait influences its expression. This could be the case when combs are regularly removed from colonies to measure post-capping durations (see Additional file 1). The regulation of hive temperature is crucial for optimal brood development [262–264] and might be disturbed, thus affecting post-capping duration.

### Matching expectations

The main expectation from a stock selected for resistance against *V. destructor* is that it efficiently (in terms of both money and honey) protects the colonies in spite of the high horizontal transmission of the parasite in typical apiaries [148, 151, 214]. Aside from resistance, selected colonies should remain productive since most beekeepers aim at collecting one or more hive products. The ‘Russian’ stock tested in Germany revealed lower honey production, smaller population size, lower calmness during inspection, and higher defensive behaviour than the local controls [235, 236, 265, 266]: as a result, the import of this stock was not recommended for German beekeepers [233].

Detrimental traits have also been shown to appear in colonies that are selectively bred for resistance to *V. destructor*. In Austrian *A. m. carnica* colonies, a positive phenotypic correlation (r = + 0.17) between the infestation level and honey production was observed. In spite of the low coefficient of the correlation, this implies that colonies producing more honey also reared more brood, which promotes mite reproduction [267]. Such trade-offs may also occur in some lineages selected for VSH with poor brood patterns [67] and may express suboptimal colony development, which would hinder honey collection or pollination ability. Similarly, although artificially uncapped-recapped worker brood have similar adult longevity compared to controls [268], negative effects on their behaviour and performance could not be excluded [109]. A possible trade-off was also observed between MNR and honey production. Colonies selected for MNR were found to be smaller than controls or hybrids [42, 269]. In a German population selected for hygienic behaviour and grooming, lower colony size, lower gentleness and slightly lower spring honey harvest were recorded compared to control colonies in various environments [270, 271].

## Perspectives towards more efficient selection

Selection against *V. destructor* is a complex process. The currently described host defence mechanisms are diverse and their genetic background is uncertain, as is their effective involvement in colony survival (Fig. 1). This is highlighted by publications reporting contrasting results on the link between traits and colony survival, as well as on the heritability of these traits and therefore on the potential for their improvement by selection. Moreover, expression of resistance traits is heavily influenced by unknown or uncontrollable environmental factors, with the consequence that local selection successes cannot be replicated elsewhere. As long as the impact of local environment on the ability of the selected traits to limit infestation remains unknown, progress towards surviving stock will likely be limited. Finally, practical limitations make selection processes tedious and limit their efficient implementation in the field. The available literature shows that proposals made 30 years ago still have to be achieved [272]. The lack of progress towards the selection of honey bee lineages surviving infestations by *V. destructor* is probably not due to the generation time of the honey bee, which is short compared to other livestock such as cattle, but is likely due to caveats in selection strategies and knowledge gaps in our understanding of resistance mechanisms.

### Improving selection strategies

Given that the traits described as conferring resistance to honey bee colonies may exhibit regionally variable efficiency in terms of improving survival or at least in limiting colony infestation (see Additional file 3: Tables S2 to S7), an a priori choice of resistance traits to be selected in any given susceptible population is a hazardous strategy to obtain surviving colonies. Recording potential resistance traits in the population targeted before initiating a selection program might help identify those that are most relevant. A further challenge to choosing relevant traits to be selected is that in all naturally-resistant populations investigated to date, various combinations of resistance mechanisms are thought to contribute to colony survival [64, 70]. Thus, a limited response to selection is expected when only one trait is selected, which seems to be a common procedure in current selection programs. Increasing the expression of a single trait to levels at which no other trait is required might lead to resistance, but this resistance may reach the biological limits of the host [273]: if workers are performing one resistance behaviour with high intensity and frequency, they may have less time to perform other tasks that are also essential to colony functioning. Selection for multiple resistance traits would thus be preferable, adding to the workload required to reach the selection objective.

Multiple solutions and perspectives have been proposed to decrease the workload associated with phenotyping. Phenotype acquisition could first be facilitated by automated devices providing reliable image-based counts of mite fall or of adult infestation rates [274], for example. Once relevant resistance traits are known, marker-assisted selection could also help rapid evaluation of the genetic values of large numbers of colonies [64]. Relying on natural selection to obtain resistance to *V. destructor* [66] could also be a way to reduce the workload associated with phenotyping since the traits leading to survival do not need to be known when only survival and ability to reproduce are the objectives [275]. However, natural selection does not consistently favour high productivity, low defensive behaviour, behaviour on comb and a low propensity to swarm [60, 66, 215, 276–279], which are often desired by beekeepers.

If they are to be adopted broadly, selected stocks must indeed fulfil beekeepers’ expectations. The extent to which decreased performance may hinder the acceptance of a resistant stock by beekeepers is poorly understood. Surveying the objectives and expectations of beekeepers [280, 281], and analysing under which circumstances *V. destructor* resistance could be favoured over profitability [281], could help define currently valued traits before initiating selection programs. A further challenge is that the desired traits may change faster than selection programs can generate the corresponding lineages, which, at best, takes several years. Several years are also necessary to take the local environmental effects on colony survival into account. Although this may delay reaching the final objective, it would make the success of the selection program more likely.

It is not sufficient to formalize a breeding goal and to optimize the selection strategy to obtain resistant populations; the host genome must also include the genes that enable this goal to be reached. Calculating heritability for the desired traits ascertains whether the observed variation has a genetic origin and therefore can be improved by selection. Heritability estimates known to date show that values for resistance traits are in the range of other desirable production or behavioural traits [282]. Thus, selected resistance traits should have contributed to balance the host–parasite relationship. Selected lineages matching some of the beekeeper criteria were obtained through relatively simple selection procedures [283–289], whereas selecting resistance to *V. destructor* seems much more challenging, suggesting that the relevance of the resistant traits should be reconsidered.

### Improving our understanding of resistance mechanisms

The choice of traits currently used in selection for resistance derives from observations of naturally-resistant colonies. However, the traits or combination of traits that provide protection to infested colonies have not been empirically determined. Thus, the role of currently used traits towards improving survival remains hypothetical. Determining the role and importance of the suspected traits is limited by the complexity of the experimental designs required. The prerequisites for such tests are honey bee lineages that express different trait combinations at different intensities so that a comparative study can be performed. The multi-year duration of the tests required due to the relatively long generation time and longevity of honey bee colonies and the time required for *V. destructor* to exert an impact on colony survival add to the challenge. In addition, data on the genetic background of resistance traits to help choose traits for selection are lacking. Although some values are available (Fig. 1) and see Additional file 2: Table S1, there is a need to obtain additional estimates from a larger number of colonies and environmental conditions to provide more reliable baseline information on the traits that may be more easily and rapidly improved by selection.

Increased international collaborations among scientists such as in the COLOSS network (http://www.coloss.org) could help develop a concerted and standardized approach to tackle the challenge of unveiling the complex mechanisms enabling colony survival [64] and environmental factors influencing their expression.

In theory, the results of ongoing selection programs could be used to fill some of these gaps, as they represent real-life tests of the hypothesis that selected traits contribute to colony survival. Unfortunately, the lack of published data on genetic progress achieved and on the associated colony survival performance (Fig. 1) make it impossible to determine whether colony survival was genetically improved or whether it was linked to favourable environmental conditions. Even in research programs, controls are often not used in parallel to the selected population to take environmental effects into account [290]. Also problematic is the fact that when genetic progress is shown for a selected trait, implications in terms of colony survival are often not presented [291], hindering our understanding of the mechanisms involved. An improved availability of information on genetic parameters linked to resistance traits, on selection strategies used, on outcomes and limitations of past and current programs could not only provide better insights into how best to improve selection programs but also help promote currently available resistant lineages [292]. A more systematic publication of such information could be achieved by promoting networks and partnerships between research institutes and honey bee breeders. Such collaborative efforts would also allow inclusion of a large number of colonies and environments and generation of more reliable data and, therefore, the development of better phenotypes, which are crucial to enable selection progress towards the survival of *A. mellifera* colonies infested with *V. destructor*. Such collaborations were often initiated, but faded away because scientific projects are funded for a limited time, whereas the efforts required must span many years. Thus, the availability of longer-term national funding schemes is desirable to ensure sustainable scientific support of breeding efforts.

### Conclusions

The urgent need to identify a sustainable solution to the *V. destructor* problem conflicts with the long lastingness of selection programs, which span years or decades. Although potentially a sustainable solution to the ‘*Varroa* problem’, the current rate of progress of programs that focus on traits promoting the survival of *V. destructor-*infested colonies suggests that the strategies followed should be critically reconsidered to ensure that goals are reached with sufficient rapidity for selection to represent a valid solution. These strategies should be rooted in better knowledge of survival mechanisms and of the environmental factors influencing them. In addition, they should be optimally designed to provide progress as rapidly and efficiently as possible in terms of both time and resources. Better program design and, more specifically, the regular assessment of selection progress are required. Failure of selection programs is likely until the current gaps in knowledge about survival mechanisms are filled; thus, the sooner shortcomings are recognized, the faster the program can be re-directed.

## Supplementary information

**Additional file 1.** Trait evaluation methods. The following traits are presented: ‘mite non reproduction (MNR)’ [71, 293–295], ‘Varroa sensitive hygiene’ (VSH) [114, 115, 141, 157, 182, 186, 256–261, 296], ‘uncapping-recapping [71, 108, 169], ‘hygienic behaviour towards dead brood’ [74, 158, 297–303], ‘reduced post-capping stage duration’ [136, 139, 304–306], ‘grooming’ [307, 308], ‘mite infestation level’ [76, 172, 309–313].

**Additional file 2: Table S1.** Published heritability estimates for resistance traits [54, 75, 139, 182, 267, 304, 314–332].

**Additional file 3: Table S2.** Link between ‘mite non reproduction’ (MNR) and colony survival or infestation level reported in literature [70, 72, 97, 106, 136, 170–172, 269, 333–345]. **Table S3.** Link between ‘Varroa sensitive hygiene’ (VSH) and colony survival or infestation level reported in literature [72, 102, 108, 109, 112, 114, 115, 169, 259, 346–348]. **Table S4.** Link between ‘uncapping–recapping’ and colony survival or infestation level reported in literature [73, 97, 117, 306]. **Table S5.** Link between ‘hygienic behaviour towards dead brood’ and colony survival or infestation level reported in literature [42, 97, 172, 183, 185, 215, 231, 270, 312, 315, 319, 320, 324, 325, 327, 328, 340, 349–366]. **Table S6.** Link between ‘reduced post-capping stage duration’ and colony survival or infestation level reported in literature [54, 74, 139, 304, 306, 312, 367–370]. **Table S7.** Link between ‘grooming’ and colony survival or infestation level reported in literature [74, 130, 131, 166, 172, 215, 261, 307, 312, 354, 360, 371–378].

## Data Availability

Not applicable.
