# Supplementary material for: Advances and perspectives in selecting resistance traits against the parasitic mite Varroa destructor in honey bees
Source: Genet Sel Evol. 2020 Nov 27;52:71. doi: 10.1186/s12711-020-00591-1 (PMC7694340; doi:10.1186/s12711-020-00591-1)
Supplement: Supplementary file 1 — Additional file 1. Trait evaluation methods. The following traits are presented: ‘mite non reproduction (MNR)’ [71, 293–295], ‘Varroa sensitive hygiene’ (VSH) [114, 115, 141, 157, 182, 186, 256–261, 296], ‘uncapping-recapping [71, 108, 169], ‘hygienic behaviour towards dead brood’ [74, 158, 297–303], ‘reduced post-capping stage duration’ [136, 139, 304–306], ‘grooming’ [307, 308], ‘mite infestation level’ [76, 172, 309–313]. [file 12711_2020_591_MOESM1_ESM.docx]

# Additional file 1 – Trait evaluation methods

**Mite Non Reproduction (MNR):** The presence of male and female offspring is, in general, recorded in infested brood cells containing worker pupae at 8–11 [293] or 7–12 days post-capping [71]. MNR is diagnosed when the foundress mites produced no offspring, only a male offspring or a dead male offspring resulting in unmated daughters, or when a delay in oviposition prevents the production of adult mated daughter mites prior to host emergence [294, 295].

**Varroa Sensitive Hygiene (VSH):** VSH has been measured in various ways. The most precise yet also time-consuming method involves measuring the removal of cells manually infested with *V. destructor* [115, 182, 256]. Less effort-intensive methods measure the changes in the infestation rate of brood frames transitorily inserted into highly infested colonies before being returned to the test colonies [157, 186, 257-260]. A further method involving the use of photographs of the inserted brood frame before and after being returned to the test colony exists [114, 261]. It allows for the determination of whether the reduction in infestation measured following the opening of remaining brood cells correlates with brood removal or not. For research purposes, the time-consuming analysis of video recordings can help to identify the workers performing the task [141, 296]. This can be useful when later genotyping the individuals involved in removal or using workers to produce drones in experimental selection programmes [296]. A comparison of the different VSH protocols is currently unavailable to help choose the best protocol, minimise costs and maximise precision.

**Uncapping-Recapping:** Several methods were used to measure the frequency of uncapping*–*recapping. Some studies recorded the percentage of removed mites without the removal of the pupae in artificial infestation experiments [169], whereas more recent studies used the absence of cocoon silk on the inner side of the cell cap as a marker for this behaviour since lack of silk indicates that the cell has been opened and resealed by workers [71, 108].

**Hygienic behaviour towards dead brood:** The degree of hygienic behaviour of a colony is measured as the removal rate of dead worker pupae by adult workers after a certain amount of time. Brood is killed either by placing a piece of brood comb in a freezer [297, 298,300], in liquid nitrogen [299] or by pinning individual brood through the wax capping with a needle [300, 301], the latter being less demanding in terms of material and time. These methods appear similarly effective in quantifying hygienic behaviour: significant positive correlations, ranging between 0.54 and 0.96, were obtained in comparative studies [158, 300, 302]. Thus, the selection output should be independent of the method used, and the ease of implementation can guide the method chosen to assess the phenotype. In Central Europe, the pin-test method was recommended to local beekeepers [303] as it was found to give more repeatable and accurate results than the other equivalent methods [74].

**Reduced post-capping stage duration:** After the sealing of individual worker cells, the duration of the post-capping stage is monitored at short time intervals either in the hive [136, 139, 304] or in an incubator [305, 306]. The frequency of these observations determines the precision of the assessment of the post-capping duration. The hive method offers the advantage of including nestmate effects and effects of the colony environment in the analysis. To the best of our knowledge, the two methods have not been compared to date, so their respective impact on the evaluation of the post-capping stage duration is not known.

**Grooming:** In the field, grooming is evaluated by sampling the mites falling from the colony onto a bottom board made inaccessible to honeybee workers with a mesh. Mites are collected and body damage such as missing legs or injured shield, is recorded [307]. The percentage of damaged mites is used as an indicator of the grooming capacity of the colony. In the laboratory, methods are developed to monitor damages of mites added to caged workers [308].

**Mite infestation level:** Several methods can be used to measure infestation levels or rates. The most precise method involves killing the entire colony [76]. In practice, even if the queen could be spared for selection purposes, this method is too costly to be applied. Alternatively, varroacide treatments can be used, although they are destructive towards mites and, therefore, prevent reliable repeated measurements. Mite counts in worker or brood samples and the recording of the natural mite fall beneath the colony are thus preferred for estimating the mite load of the colony [76], and they are employed as selection traits in beekeeping conditions [309-311]. The mite population growth rate, for instance, between spring and late summer, is also used as a selection trait: in research programmes, lineages with either low or high infestation growth rates were selected [172, 312, 313], for example, to determine the mechanisms linked to the observed infestation differences [172, 312]. Similar approaches could also be employed in beekeeping conditions to select for lineages with lower infestation.
