# Supplementary material for: Advances and perspectives in selecting resistance traits against the parasitic mite Varroa destructor in honey bees
Source: Genet Sel Evol. 2020 Nov 27;52:71. doi: 10.1186/s12711-020-00591-1 (PMC7694340; doi:10.1186/s12711-020-00591-1)
Supplement: Supplementary file 3 — Additional file 3: Table S2. Link between ‘mite non reproduction’ (MNR) and colony survival or infestation level reported in literature [70, 72, 97, 106, 136, 170–172, 269, 333–345]. Table S3. Link between ‘Varroa sensitive hygiene’ (VSH) and colony survival or infestation level reported in literature [72, 102, 108, 109, 112, 114, 115, 169, 259, 346–348]. Table S4. Link between ‘uncapping–recapping’ and colony survival or infestation level reported in literature [73, 97, 117, 306]. Table S5. Link between ‘hygienic behaviour towards dead brood’ and colony survival or infestation level reported in literature [42, 97, 172, 183, 185, 215, 231, 270, 312, 315, 319, 320, 324, 325, 327, 328, 340, 349–366]. Table S6. Link between ‘reduced post-capping stage duration’ and colony survival or infestation level reported in literature [54, 74, 139, 304, 306, 312, 367–370]. Table S7. Link between ‘grooming’ and colony survival or infestation level reported in literature [74, 130, 131, 166, 172, 215, 261, 307, 312, 354, 360, 371–378]. [file 12711_2020_591_MOESM3_ESM.docx]

# Additional file 3 – Link between resistance trait expression and colony survival or infestation level reported in literature.

Publications are classified as indicating apparent or no link between the resistance traits and colony *Varroa destructor* infestation level and between traits and colony survival. ‘Apparent’ is used in case of a positive link with the target parameter since no empirical study has formally demonstrated a causal effect. As strength and direction of the links may vary depending on the environment/population considered, countries/regions in which investigations took place are indicated. This also enables finding references corresponding to local needs of selection programmes. When the observations were made in naturally resistant population, this is mentioned. In all other cases, the stocks investigated were originally susceptible.

## Additional file 3 Table S2 : Mite Non Reproduction (MNR).

| **colony infestation level** | |
| --- | --- |
| *Apparent link* | *No link* |
| - in colonies selected for low infestation growth rates for 16 weeks, Canada [333] - in an *A. m. carnica* population, Central Europe: [97]. - colonies selected for MNR showed lower infestation rate than other lines, including colonies of surviving ‘Russian’ stock, USA [171]. - colonies headed by MNR and hybrid queens mated with unselected drones had lower infestation levels when compared to the controls, USA [269, 334]. - Colonies of the ‘Russian’ stock, less infested than controls, expressed MNR [335, 336]. | - in colonies from various European origins tested in Germany [337]. - in an *A. m. ligustica* population subjected to diverging selection for high and low infestation levels, Italy [172]. - only a third of the variation in infestation growth rate attributed to the proportion of fertile mites together with the ratio of mites infesting brood versus adults, USA [106]. - environmental factors rather than genetic host effects explained the variations in mite reproduction, USA [338]. - Crosses between MNR selected lineages and lineages selected for hygienic behaviour resulted in lower infestation rates in the hybrids when compared to the colonies selected for hygienic behaviour [339, 340] but mite fertility or the number of viable offspring produced did not differ, USA [340]. |
| **colony survival** | |
| *Apparent link* | *No link* |
| In naturally surviving honey bees:   - Africanized honey bees, Brazil [136, 341]. - Avignon [70], Toulouse [342], France - Norway [72]. - *A. m. scutellata*, South Africa [343]. - Gotland and hybrids with local susceptible stock, Sweden [344]. | Fertility and fecundity of local mites did not differ in:   - naturally surviving Gotland colonies, colonies headed by daughters from USA MNR queens vs. local *A. m. carnica* colonies, Germany [170]. - colonies headed by F2 Gotland vs. *A. m. mellifera*, *A. m. carnica* or *A. m. ligustica* queens, Germany [345]. |

## Additional file 3 Table S3: Varroa Sensitive Hygiene (VSH)

| **colony infestation level** | |
| --- | --- |
| *Apparent link* | *Insufficient link* |
| - workers tended to better uncap single-infested cells in low than in high infestation colonies, France [346]. - removal of infested brood observed [102], Germany - colonies selected for VSH removed significantly more mites from foreign combs than controls, USA [108, 109, 259]. - in colonies of Russian line, the removal of infested brood is suggested as the main reason for reduced infestation levels when compared to controls [114]. A negative correlation (r = -0.25) was measured between removal of infested brood and colony infestation level, USA [112]. | - colonies with high VSH ability display a higher removal of infested brood in the case of multiple than single infestations [102], Germany. |
| **colony survival** | |
| *Apparent link* | *No link* |
| - in naturally surviving Africanized honey bees, Brazil [169, 347]. - in naturally-surviving *A. m. scutellata* in South Africa [348]. - in a population selected for colony survival, The Netherlands [115]. | - naturally surviving population, Norway [72] |

## Additional file 3 Table S4: uncapping*–*recapping

| **colony infestation level** | |
| --- | --- |
| *Apparent link* | *No link* |
| - in colonies of various origins in Europe and the USA [97, 117]. |  |
| **colony survival** | |
| - *Apparent link* | *No link* |
| *Identified in several naturally surviving populations*   - *colonies of European origins* more frequently uncapped and recapped infested cells than local susceptible colonies, France, Norway and Sweden [306] - African and Africanized populations of South Africa and Brazil expressed higher levels of recapping, than uninfested populations in *Australia* and the *United Kingdom* [73] |  |

## Additional file 3 Table S5: hygienic behaviour towards dead brood

| **colony infestation level** | |
| --- | --- |
| *Apparent link* | *No link* |
| - in naturally surviving Africanised honey bees, Brazil [324, 349, 315]. - in naturally surviving *A. m. simensis,* Ethiopia [350] - naturally surviving *A. m. scutellata,* as well as neighboring subspecies studied in Kenya [351]. - in colonies of European origin established in Canada [327, 352]. - in *A. m. carnica,* Arbeitsgemeinschaft Toleranzzucht [AGT, [www.toleranzzucht.de](http://www.toleranzzucht.de)] population, Germany [319, 320, 353]. - lower infestation rates in colonies selected for hygienic behaviour and grooming compared to controls, Germany [270, 354]. - United Kingdom [355]. - ‘Russian’ colonies showed higher hygienic behaviour than control colonies, USA [356]. - Minnesota colonies bred for hygienic behaviour showed lower infestation than controls, USA [357]. - in colonies selected for hygienic behaviour, USA [42]. | - in colonies of European origin selected for hygienic behaviour, Argentina [358]. - in unselected colonies, Belgium [183]. - in unselected colonies, Chile: [359]. - in *A. m. carnica*, Germany [97]. - in *A. m. ligustica* colonies, Italy [172]. - in *A. m. mellifera* and *A. m. carnica* colonies, Switzerland [328]. - in two populations bred for survival, The Netherlands [216, 360, 361]. - in colonies bred for hygienic behaviour against dead brood, USA [185, 362]. - in colonies pre-selected for various resistance traits, USA [312]. |
| **colony survival** | |
| *Apparent link* | *No link* |
| - untreated colonies selected for hygienic behaviour better survived in the presence of *V. destructor* when compared to unselected colonies, Canada [325]. | - No significant differences in survival between the lineages selected for removal of pin-killed brood and unselected lineages when left untreated, Argentina [363]. - Colonies from the AGT and Kirchhain populations selected for hygienic behaviour did not show better survival when tested in different environmental conditions in the absence of mite control when compared with mostly unselected colonies of various origins, Germany [231]. - in naturally surviving Africanised honey bees, Mexico [360, 364]. - in naturally surviving colonies from the Gotland population, Sweden [215]. - *A. m. anatoliaca*, no better survival of hygienic over non-hygienic colonies with initially equalised mite loads, Turkey [365]. - in probably unselected colonies, United Kingdom, England [355]. - in Minnesota colonies bred for hygienic behaviour late season varroacide treatments still needed to prevent winter losses, USA [340, 366]. - in a population selected for hygienic behaviour, no significant difference for the time of year at which the treatment threshold was reached compared to unselected controls, USA [42]. |

Additional file 3 Table S6: reduced post-capping stage duration

| **colony infestation level** | |
| --- | --- |
| *Apparent link* | *No or weak link* |
| - in different strains of European *A. mellifera* colonies, Germany [139, 304]. | - variations in the post-capping stage duration (270 to 300 h) unlikely to have an effect on the infestation level, Germany [74]. - in colonies with *A. m. capensis* ancestry selected for the reduced post-capping stage, the link [367] lost significance in the following generation. No significant difference in colony infestation rates compared to controls, Poland [368]. - in colonies pre-selected for various resistance traits, USA [312]. |
| **colony survival** | |
| *Apparent link* | *Unlikely link* |
| - Identified in naturally-surviving *A. m. scutellata* and *A. m. capensis*, South Africa [54, 369, 370] | - in a naturally surviving population, Norway [306]. |

Additional file 3 Table S7: grooming

| **colony infestation level** | |
| --- | --- |
| *Apparent link* | *No link* |
| - in *A. m. carnica* colonies, Austria [371]. - colonies selected for low *V. destructor* infestation growth rates show a higher proportion of damaged mites compared to lines selected for high infestation growth rates [372]. - in overwintering colonies expressing high grooming behaviour, daily mite mortality in experimental cages was higher than a colony with low grooming, Canada [131]. - *A. m. carnica* colonies with a high rate of damaged mites had a lower increase in their mite population than colonies with a low rate of damaged mites, Germany [74, 130, 354]. - in naturally surviving Africanised honey bees, Mexico [360, 373]. - colonies selected on the rate of damaged mite and tested in different beekeeping conditions showed lower mite populations than controls in the majority of the test locations, USA [374]. | - in naturally surviving Africanised honey bees, Mexico [372]. - *A. m. carnica* colonies selected for low infestation growth rates in Austria [307] showed higher infestation rates and less damaged mites than unselected *A. m. carnica* lineages when tested in Germany [375]. - in ‘Russian’ stock, Canada [372] - in a population selected for either low or high *V. destructor* population growth rates, Italy [172]. - in colonies pre-selected for various resistance traits, USA [312]. |
| **colony survival** | |
| *Apparent link* | *No link* |
| - in *A. m. intermissa* colonies, Tunisia [166, 376]. - Lower *V. destructor* infestation combined with increased colony survival and the increased expression of a grooming-related gene were identified in a population selected for grooming compared to a susceptible control population [377]. | - in a naturally surviving population, Norway [261] - in naturally surviving colonies from the Gotland population, Sweden [215]. - in two populations selected for survival, The Netherlands [378] |
